# Gene expression dynamic analysis reveals co-activation of Sonic Hedgehog and epidermal growth factor followed by dynamic silencing

**DOI:** 10.18632/oncotarget.27547

**Published:** 2020-04-14

**Authors:** Vahed Maroufy, Pankil Shah, Arvand Asghari, Nan Deng, Rosemarie N.U. Le, Juan C. Ramirez, Ashraf Yaseen, W. Jim Zheng, Michihisa Umetani, Hulin Wu

**Affiliations:** ^1^Department of Biostatistics and Data Science, School of Public Heath, University of Texas Health Science Center at Houston, Houston, TX, USA; ^2^Department of Epidemiology, Human Genetics and Environmental Sciences, School of Public Health, University of Texas Health Science Center at Houston, Houston, TX, USA; ^3^Center for Nuclear Receptors and Cell Signaling, Department of Biology and Biochemistry, University of Houston, Houston, TX, USA; ^4^Facultad de Ingeniería de Sistemas, Universidad Antonio Nariño, Bogota, Colombia; ^5^School of Biomedical Informatics, University of Texas Health Science Center at Houston, Houston, TX, USA; ^6^HEALTH Research Institute, University of Houston, Houston, TX, USA; ^*^These authors contributed equally to this work

**Keywords:** pathway cross-talk, gene expression dynamics, gene regulatory network, Sonic Hedgehog, epidermal growth factor

## Abstract

Aberrant activation of the Sonic Hedgehog (SHH) gene is observed in various cancers. Previous studies have shown a “cross-talk” effect between the canonical Hedgehog signaling pathway and the Epidermal Growth Factor (EGF) pathway when SHH is active in the presence of EGF. However, the precise mechanism of the cross-talk effect on the entire gene population has not been investigated. Here, we re-analyzed publicly available data to study how SHH and EGF cooperate to affect the dynamic activity of the gene population. We used genome dynamic analysis to explore the expression profiles under different conditions in a human medulloblastoma cell line. Ordinary differential equations, equipped with solid statistical and computational tools, were exploited to extract the information hidden in the dynamic behavior of the gene population. Our results revealed that EGF stimulation plays a dominant role, overshadowing most of the SHH effects. We also identified cross-talk genes that exhibited expression profiles dissimilar to that seen under SHH or EGF stimulation alone. These unique cross-talk patterns were validated in a cell culture model. These cross-talk genes identified here may serve as valuable markers to study or test for EGF co-stimulatory effects in an SHH+ environment. Furthermore, these cross-talk genes may play roles in cancer progression, thus they may be further explored as cancer treatment targets.

## INTRODUCTION

It is now well known that, in most cancer patients, there are multiple genetic aberrations and deregulation of multiple signaling pathways that work in a synergistic manner to initiate and promote the tumor. Studying the cooperation between these oncogenic pathways could help identify genes commonly regulated by oncogenic pathways and, importantly, genes that are synergistically regulated, the latter of which are likely to play important roles in tumor-initiation and cancer growth. These genes might then be exploited for novel therapeutic approaches. Although most studies based their data analysis on analysis of covariance (ANOVA) or classic regression models, we developed an alternative comprehensive approach that exploits Ordinary Differential Equations (ODE) models and gene regulatory network analysis [1–3]. This model is able to take into account the dynamic and temporal behavior of genes and learn the dynamic relationship between genes.

To validate our approach, we selected Sonic Hedgehog (SHH) and epidermal growth factor (EGF) signaling pathways. The Hedgehog signaling pathway is primarily involved in tissue development. However, in adults, the activation of this signaling pathway has been implicated in cancers of the brain, including medulloblastoma, lung, prostate, breast, and most notably in the skin (basal cell carcinoma) [4]. Dysregulation of this pathway has been shown to promote stem cell proliferation and tumor initiation [5]. Other important proteins in this pathway, Smoothened and Patched homolog 1, encoded by *SMO* and *PTCH1*, respectively, in turn, regulate the expression of the *GLI1* gene. *GLI1* encodes for the zinc finger transcription factor Glioma-associated oncogene homolog 1 (GLI1)–the main effector of SHH pathway [5]. Inhibition of the SHH pathway by targeting these transcription factors and their receptors could lead to a cure for many malignancies. For example, Vismodegib, an antagonist of Smoothened, is approved for the treatment of basal cell carcinoma [6]. In addition, the upstream activators of the SHH pathway are dependent on EGF- receptor-mediated activation of the RAS/RAF/MEK/ERK pathway, but not of the PI3K/AKT pathway [7]. Synergistic integration of SHH and EGF signaling has been identified as a critical step in oncogenic transformation, neural stem cell proliferation [8], and development of tumor types such as skin, prostate, pancreas, and basal cell carcinoma [9–13]. The combination of SHH and EGF leads to a synergistically activated growth response in basal cell carcinoma and tumor-initiating pancreatic cancer cells both *in vitro* and *in vivo* [9]. Activation of at least one of these pathways is implicated in one-third of all cancers [14–16]. Although this synergy has been studied, details of interactions at the molecular level are not well known.

In this study, we used publicly available gene expression profiling datasets from a human medulloblastoma cell line that possesses a fully inducible endogenous SHH pathway according to gene expression profiling [8]. Briefly, cells were subject to four different experimental conditions: i) no stimulation control (CTRL), ii) EGF stimulation only (EGF+), iii) Sonic Hedgehog stimulation only (SHH+), and iv) co-activation of EGFR and Hedgehog (EGF+SHH+). The cells were harvested for gene expression profiling at 14 different time-points over a 24-hour period after cell stimulation. In the original study, an Illumina HumanHT microarray was used to obtain the gene expression data. It included expression values for 47,231 probes. ANOVA was used and implemented on fold-change ratios for identifying the target genes affected by synergy between SHH and EGF signaling. Specifically, linear regression models were fitted to fold-change ratios over time, and experimental conditions served as covariates. A total of 3,827 genes (from 4,580 probes) were determined to be significantly expressed in all pairwise comparisons of treatment conditions. However, only 12 genes with a documented role either in EGF or SHH pathways were used for further validation, and under co-activation of both pathways, canonical SHH target genes such as *GLI1*, *PTCH*, and *HHIP* were downregulated, while selected EGF target genes such as *MMP7*, *VEGFA*, and *IL8* were synergistically upregulated [8].

Here, we used a series of analytic and modeling approaches to explore the dynamic activities of the entire gene population. Genes with significant dynamic responses were identified, and those that follow similar patterns over time were grouped with a clustering algorithm and then validated in a cell culture model. The interactive relationship between genes as stimulators or inhibitors of each other was explored through the regulatory network analysis. We also looked at the influence of the synergistic co-activation of the EGF and SHH pathways on individual gene levels and the overall effect at the gene network level.

## RESULTS

### EGF stimulation dominates gene regulation over SHH stimulation

Our analysis identified 7,176 genes as “dynamic response genes (DRG)” under the CTRL condition, meaning that they showed significant changes in their expression levels over time under the CTRL condition. Under the SHH+ condition, there were 50% more DRGs (10,770 DRGs) than in CTRL, reflecting the effect of SHH stimulation. The effect of EGF stimulation was stronger, leading to a 118% increase in the number of DRGs (15,659 DRGs) over CTRL. These findings imply that the downstream cascading effects of EGF stimulation activate far more genes than SHH stimulation. The EGF+SHH+ stimulation recruited the highest number of genes of the four conditions: 17,972 DRGs. This was an increase of 150% over CTRL. However, this number was less than the additive effect of SHH+ and EGF+, giving us the first evidence of some commonality or “cross-talk” between the downstream effects of stimulating the Hedgehog and EGF pathways. As much as one-third of the SHH+ downstream effects were also seen after EGF+ stimulation. Our further analysis aimed to explore this synergism and the genes that play a differential role under the synergistic condition.

### Gene response modules under EGF stimulation are synchronized into fewer expression patterns than SHH stimulation

DRGs were grouped into gene response modules (GRM) according to similar patterns of expression over time. However, to compare the number and characteristics of the GRMs between different conditions, we restricted our further analysis to only the top 3,000 DRGs on the basis of the F-ratios in each condition. This eliminated the confounding effect of the considerably larger number of DRGs under the EGF+ and EGF+SHH+ conditions as compared to the CTRL. Under the CTRL condition, the dynamic genes were responding to a wide array of homeostasis signals and physiological demands in the cells, which varied in terms of their temporality and resulted in a large number of distinct patterns of expression–namely 216 different GRMs (Table 1). Under SHH stimulation, the number of GRM patterns was close to that of CTRL. It is likely that SHH exerts its influence through a narrower set of DRGs, as there was no evidence of synchronized expression as compared to CTRL. In contrast, under EGF stimulation, the number of GRMs was as small as 48, indicating that the DRGs were synchronized into fewer expression patterns in response to the external stimulus. Under EGF+SHH+ we had almost the same number of expression patterns. This indicates that EGF must have a stimulating effect on a wider set of DRGs, which synchronizes their response, and that EGF was the dominant stimuli in the combined stimulation condition.

**Table 1:** Number of GRMs

Condition	# GRMs	# *GRMs* > 70	# genes GRM1
CTRL	216	6	617
SHH	237	6	752
EGF	48	6	952
EGF-SHH	51	7	975

The first and second columns present number of GRMs based on the first 3,000 DRGs, and number of large size GRMs, respectively, for under each condition. Third column presents the number of genes in the largest GRM under each condition.

**Figure 1:**
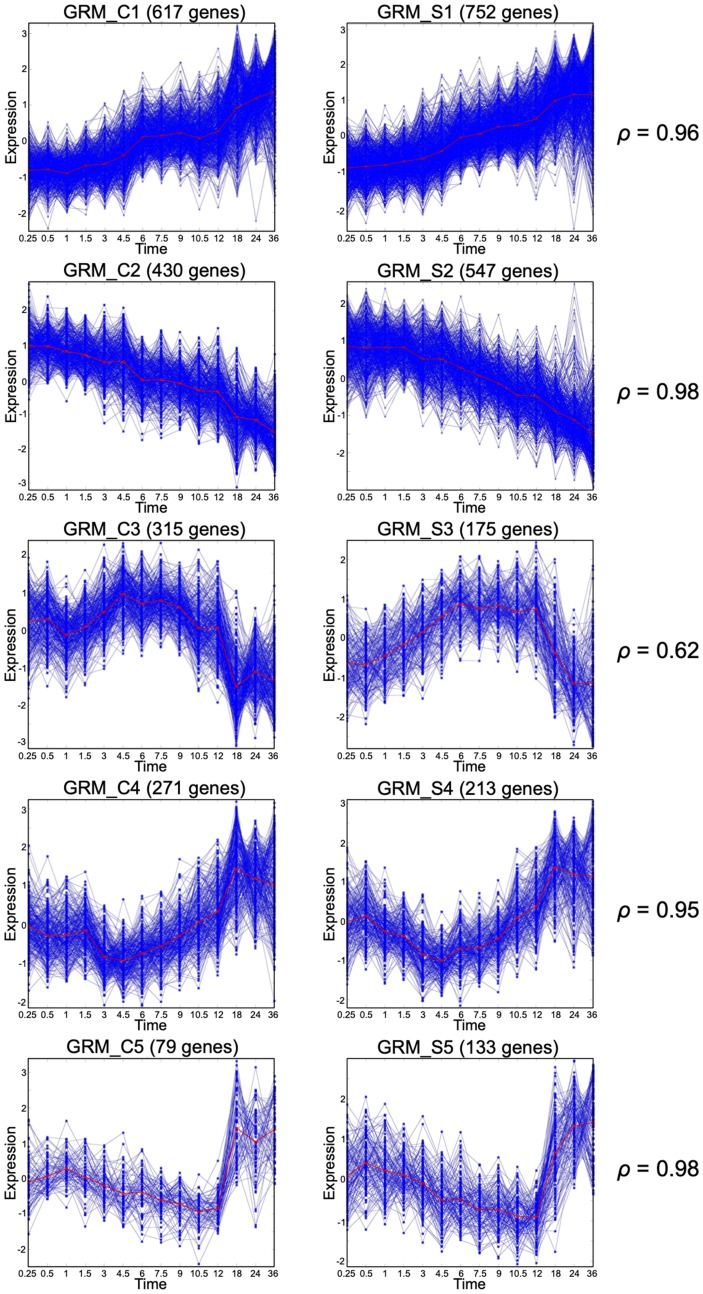
Gene response modules under Control and SHH+ conditions. Matching large size GRMs under Control (left column) or SHH+ (right column). The Spearman correlation between the mean curves (red curves) is also reported for each pair.

**Figure 2:**
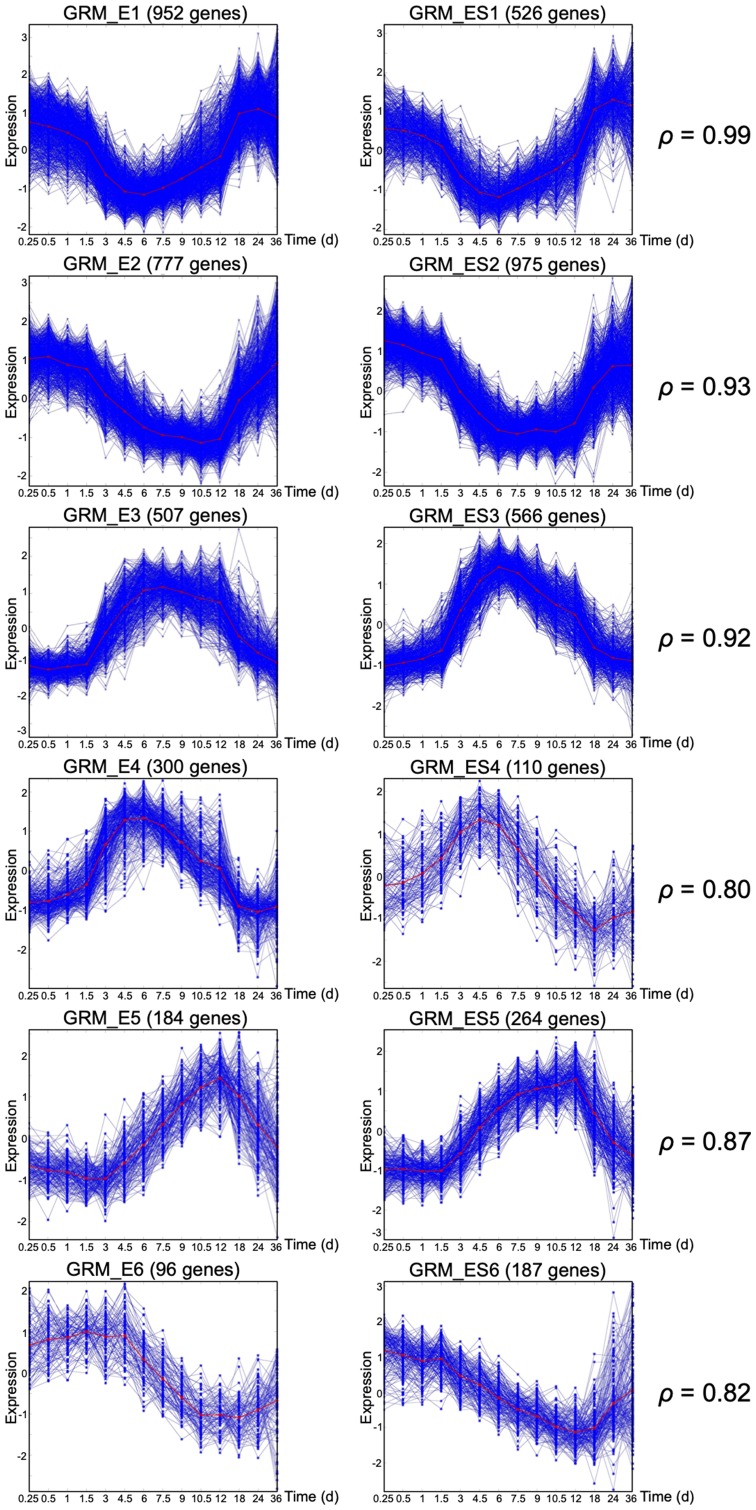
Gene response modules under EGF+ and EGF+SHH+ conditions. Matching large size GRMs under EGF+ (left column) or EGF+SHH+ (right column). The Spearman correlation between the mean curves (red curves) is also reported for each pair.

Previously, we discussed the importance of large size GRMs over small size GRMs [1]. The large size modules (LS-GRM) are related to performing various fundamental biological functions and have a consistent pattern across individuals under the same experimental condition representing the state-transition patterns in response to the stimuli. On the other hand, small-sized and single-gene GRMs tend to perform homeostasis activities and have an oscillatory temporal response. Here, we defined an LS-GRM as a GRM with 70 or more DRGs; each of the experimental conditions had at least 6 LS-GRMs while EGF+SHH+ co-stimulation resulted in seven LS-GRMs (Table 2). We performed pathway analysis and functional classification by using the DAVID functional annotation clustering tool on the 5 major LS-GRMs in the CTRL group (Figure 1). Each GRM contained genes enriched for the following GO terms: GRM_C1, cell signaling and transcriptional factors; GRM_C2, cell proliferation/division; GRM_C3, cell cycle and DNA replication; GRM_C4, cell function and transcriptional factors; and GRM_C5, stress responses (Supplementary Figure 1). We paired these LS-GRMs between conditions (Figures 1 and 2). The patterns of the large modules under CTRL paired well with those of SHH+ (Figure 1), while those under EGF+ matched with EGF+SHH+ (Figure 2). Thus, SHH+ had a marginal effect on altering overall baseline expression patterns seen under the CTRL condition and exerted its effect through a narrow subset of the gene population. In contrast, EGF+ drastically altered the expression profile of the gene population and had the predominant effect when both EGF and SHH receptors were stimulated. We further examined the number of genes that clustered into these LS-GRMs. Under EGF+ and SHH+EGF+, the first six LS-GRMs included 94% and 89% of the 3,000 DRGs, while under SHH+ and CTRL, they had 63% and 59% of the DRGs, respectively (Table 2).

**Table 2:** Number (%) of DRGs out of 3000, clustered into the major GRMs

	First 6	First 8	First 12
EGF	2,816 (0.94)	2,884 (0.96)	2,936 (0.98)
EGF-SHH	2,668 (0.89)	2,847 (0.95)	2,915 (0.97)
SHH	1,893 (0.63)	1,984 (0.66)	2,135 (0.71)
CTRL	1,782 (0.59)	1,899 (0.63)	2,066 (0.69)

First column gives the number (%) of DRGs clustered into the first 6 GRMs (the largest 6 GRMs) under each condition. The last two columns include similar numbers (%) for the first 8 and first 12 GRMs, respectively.

### EGF stimulation dominates over SHH in gene-to-gene comparisons

**Figure 3:**
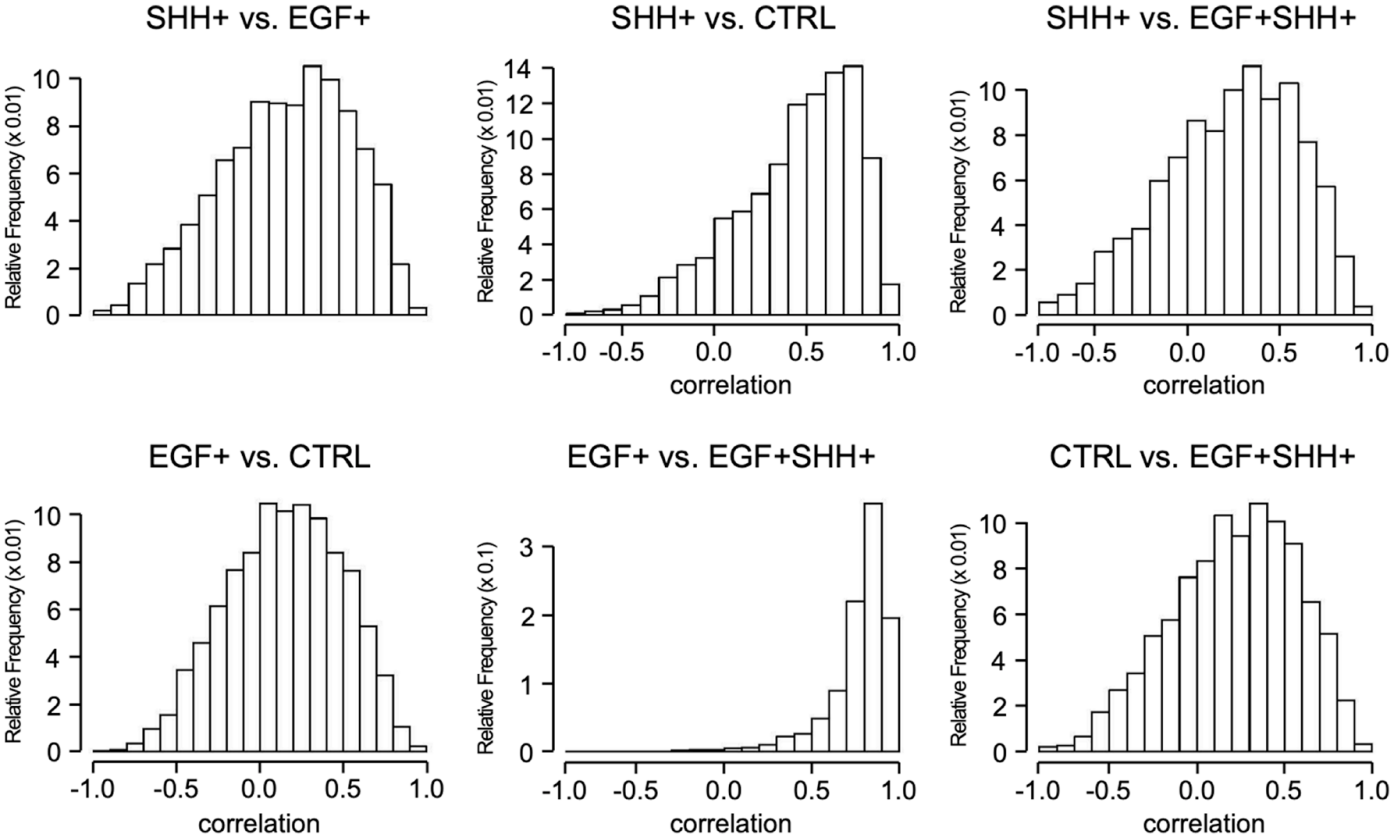
Spearman correlation of DRGs. Each panel shows the distribution of the Spearman correlations for the 3,000 DRGs under the first conditions (in panel title) with the same 3,000 genes under the second condition (in panel title).

The histograms for Spearman’s correlation of the top 3,000 DRGs under each condition were compared with the same genes under other conditions (Figure 3). A left-skewed histogram indicates a preponderance of genes behaving identically under the two experimental conditions, while a symmetric (normal) histogram indicates a preponderance towards modified expression of most genes. The SHH+ vs CTRL histogram was left-skewed and more preponderant compared with the EGF+ vs CTRL histogram. Thus, even at the gene level (gene-to-gene comparison), we found that SHH exerts its influence by modifying the expression of a far smaller set of genes than EGF. The distribution under co-stimulation was only marginally left-skewed compared to the CTRL and SHH stimulation, but extremely left-skewed when compared to EGF only, showing that, under the combined stimuli, most genes behave differently than in CTRL and SHH only stimulations, but behave similarly to the EGF only condition. Thus, stimulating SHH in an already EGF+ condition does not significantly change the expression of most DRGs. However, expression of the gene population under the SHH+ condition was greatly altered by the addition of EGF, thereby significantly altering the pathophysiology of the cell. These findings were further confirmed by comparing the number of common DRGs and genes with similar or dissimilar time-course behaviors between SHH+/EGF+SHH+ and EGF+/EGF+SHH+ conditions. Among the top 3,000 DRGs under SHH+, only 23% were overlapped with EGF+SHH+; in contrast, 54% of the top 3,000 DRGs under EGF+ were overlapped with EGF+SHH+. In addition, among the 3,000 top DRGs under SHH+, only 9% had similar gene expression behaviors under EGF+SHH+ condition (*p* > 0.7); 78.8% of the top 3,000 DRGs under EGF+ had similar behaviors under EGF+SHH+ condition (Table 3).

**Table 3:** The number (%) of genes behaving similarly (ρ>0.7) and those behaving differently (ρ<−0.7) out of 3,000 DRGs

		ρ>0.7(%)	ρ<−0.7(%)	−0.1<ρ<−0.1(%)	Other (%)
I	SHH+ vs CTRL	742 (24.7)	2 (0.1)	261 (8.7)	1,995 (66.5)
	SHH+ vs EGF+	238 (7.9)	17 (0.6)	482 (16.1)	2,263 (75.4)
	SHH+ vs EGF+SHH+	260 (8.7)	17 (0.6)	469 (15.6)	2,254 (75.1)
II	CTRL vs EGF+	182 (6.1)	19 (0.6)	541 (18)	2,258 (75.3)
	CTRL vs SHH+	736 (25.4)	1 (0)	262 (8.7)	2,001 (66.7)
	CTRL vs EGF+SHH+	233 (7.8)	16 (0.5)	479 (16)	2,272 (75.7)
III	EGF+ vs SHH+	160 (5.3)	15 (0.5)	526 (17.5)	2,299 (76.6)
	GF+ vs CTRL	136 (4.5)	15 (0.5)	566 (18.9)	2,283 (76.1)
	EGF+ vs EGF+SHH+	2339 (78)	1 (0)	25 (0.8)	635 (21.2)
IV	EGF+SHH+ vs CTRL	172 (5.7)	14 (0.5)	512 (17.1)	2,302 (76.7)
	EGF+SHH+ vs EGF+	2,356 (78.5)	0 (0)	18 (0.6)	626 (20.9)
	EGF+SHH+ vs SHH+	182 (6.1)	14 (0.5)	538 (17.9)	2,265 (75.5)

First three rows (block I) compare the trend of 3,000 DRGs under SHH+ with their trend under CTRL, EGF+, and EGF+SHH+, respectively. The latter blocks (II, III, IV) present similar numbers based on comparing the 3,000 DRGs under CTRL, EGF+, and EGF+SHH+ with the same genes under other three conditions.

### EGF stimulation increases regulatory activity

The network analysis of our analytic pipeline [3] showed that EGF+ causes the gene network to become more dense and interrelated, increasing the interaction between different GRMs and thereby providing greater regulation of the gene population’s expression (Table 4). The same thing happened when EGF was added to the SHH+ condition. As described in Section 2.1, EGF+ induced twice as many genes to become dynamic as compared to CTRL or SHH+ and altered their expression patterns. Furthermore, it reduced the overall number of independent expression patterns that the genes may take. This would require EGF to not only affect a large number of genes but also for those genes to have considerable interactions with one another so that their expression becomes harmonized in response to a single stimulus. Moreover, the network analysis showed a possible mechanism for how this was achieved. EGF+ created a far denser and interconnected network. EGF stimulation’s downstream cascading effects activated many more genes, and these genes, in turn, regulated each other to synchronize their expression dynamically over time in response to the original stimulus. Furthermore, this possible mechanism clarified the network-wide effect of how EGF+ effects dominate the SHH+ effects, even in genes that were common to both pathways.

**Table 4:** The network statistics: mean clustering coefficient and density for the networks under each condition

Condition	mean Cl coeff	Density
EGF	0.385	0.442
EGF-SHH	0.380	0.487
SHH	0.297	0.174
CTRL	0.288	0.198

Clustering coefficient measures how the neighboring GRMs are interconnected, and density shows the proportion of potential regulating edges among the GRMs.

### Transcription factors are included in cross-talk between SHH and EGF

One of the main purposes of this study was to identify genes that exhibited unique expression patterns after co-stimulation with SHH and EGF as compared to stimulation with SHH or EGF alone. These genes showed agonistic or antagonistic behavior with temporal patterns different from the initial pattern observed under either of the single stimulation conditions. We extracted two groups of genes, cross-talk genes and co-activation genes, from the list of DRGs. The cross-talk genes are those that were differentially expressed under SHH+ or EGF+ compared to CTRL; however, they exhibited completely different expression patterns under the SHH+EGF+ condition. Co-activation genes were defined as genes that were not initially differentially expressed under the individual stimuli, but were differentially expressed under combined stimuli. The mean curves of the GRMs were used to identify GRMs that exhibit cross-talk expression patterns and those that exhibit co-activation patterns. We further checked the expression patterns of genes in these GRMs to ensure that they also exhibited the same respective expression patterns (Supplementary Figure 4). We considered all 10,770 DRGs under SHH+, returning 455 distinct GRMs. To identify GRMs crucial to the gene regulatory network, the top 20 important GRMs for each network criteria were selected (Table 5). They can be referred to as GRMs that regulate most GRMs, are regulated by most other GRMs, or are most frequent intermediaries in GRM regulation pathways. These GRMs, “important GRMs,” were chosen on the basis of out-degree, in-degree, and betweenness values, respectively, as characteristics of the regulatory network.

**Table 5:** Important GRMs: obtained as the set of first 20 GRMs with highest in-degree (I), out-degree (O) and betweenness (B) coefficients

GRM	Importance	GRM	Importance	GRM	Importance
M119	O, B	M278	O	M384	I
M124	O	M290	B	M392	O
M133	O, B	M300	I	M404	I, B
M142	I, B	M307	I, B	M407	I
M156	O	M319	O, B	M408	O
M168	B	M322	O, B	M414	O
M178	O	M326	I, B	M416	I
M189	O, B	M330	I	M422	I, B
M19	O, B	M331	O	M431	I
M198	B	M337	O	M432	O
M225	B	M362	I	M436	O
M235	I, B	M37	B	M438	I, O
M244	B	M372	I, B	M454	I
M250	O	M374	I, B	M455	I
M265	O	M375	I	M72	I

In-degree (Out-degree) shows the number of GRMs regulating (regulated by) the GRM of interest. Betweenness shows the number of time that a GRM is a bridge between two other GRMs.

**Figure 4:**
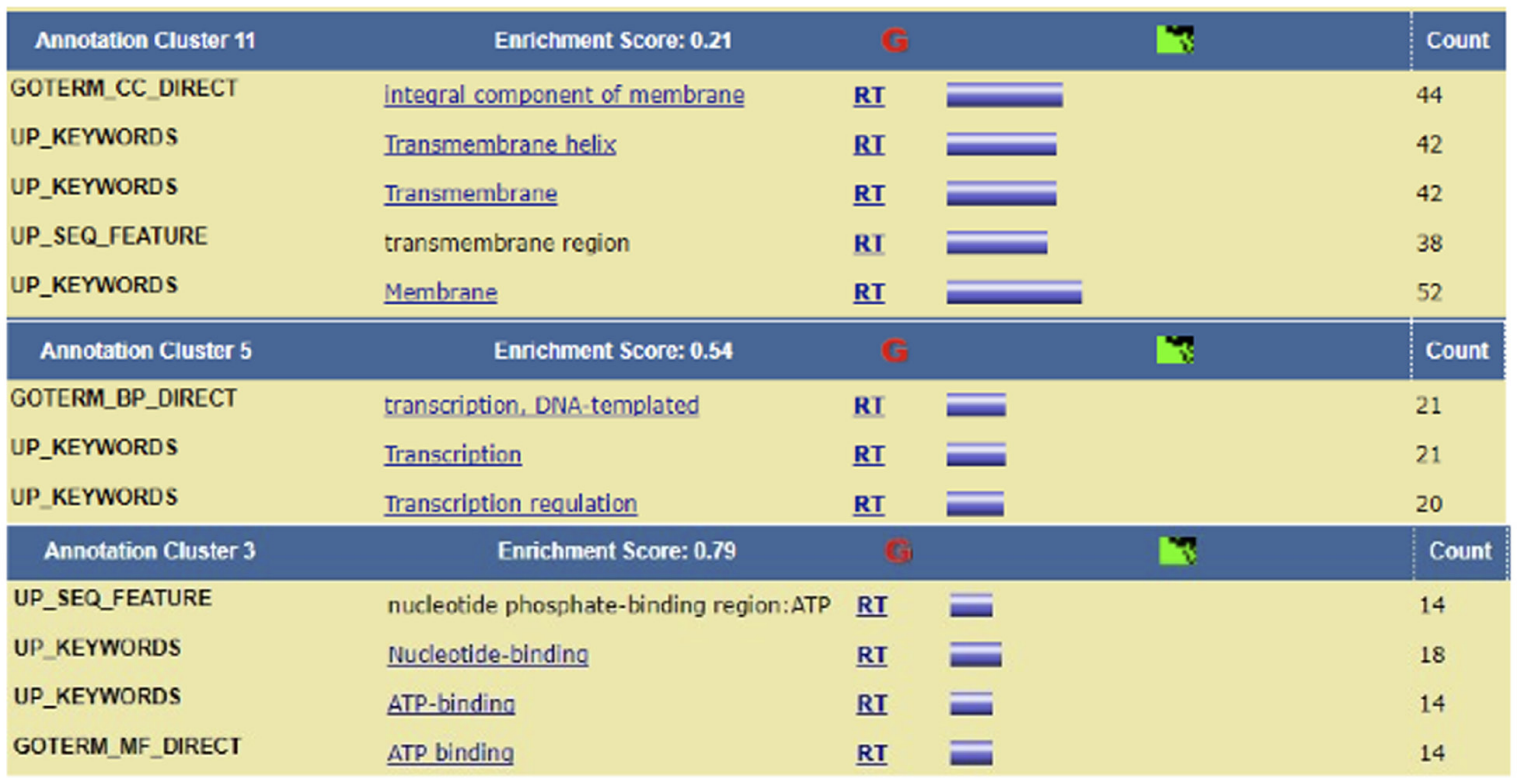
Enrichment cluster analysis of the cross-talk genes. Enrichment cluster analysis of cross-talk genes showed up-regulation of transcription-related pathways.

The genes identified as DRGs included ones already known to be in the Hedgehog pathway, namely *BCL2*, *BMP2*, *BMP4*, *CSNK1E*, *DISP2*, *FBXW11*, *FKBP8*, *GAS1*, *HHIP*, *MTSS1*, *NPC1*, *OTX2*, *PRKACB*, *PTCHD1*, *RAB23*, *SHH*, *SUFU*, *WNT1*, *WNT5B*, *WNT7B*, *WNT8B*, and *WNT9B*, indicating the validity of our approach. Among these genes, *SHH* was identified as both a cross-talk gene and a network-important gene. Although screening the GRMs and genes according to co-activation criteria did not retain any genes, 160 GRMs (out of the 455 GRMs), which comprised 179 genes, exhibited a cross-talk expression pattern that included transcription factors and proinflammatory factors, such as *TLX1* and *IL25*, respectively (Table 6). TLX1 is required for organogenesis of the spleen and neuronal cell fates and is linked to leukemia [17]. Cytokine IL-25 is a proinflammatory factor and induces NF-kappaB and IL-8 expression [18]. The detailed functional annotation is available in Figure 4.

**Table 6:** List of 179 cross-calk genes

ABCD4	CHD8	GAD2	NECAP2	SERP1
ABHD9	CHTF8	GDE1	NFIC	SERPINB7
AGGF1	CLEC4A	GRIP2	NLRP1	SHH
AHRR	CNKSR3	HHLA2	NLRP12	SIGLEC16
ALDH1A1	CNPY2	HIST1H4J	NPS	SKI
ALOX12P2	COMT	HSD11B1	NRG2	SLC7A9
AMAC1L1	CRISP1	HSPC072	OPA3	SLC9A6
AMELX	CS	HTA	OR2T3	SNORA23
ANPEP	CSF1	IFNA2	OR5H14	SNORA26
APLF	CUEDC1	IFT80	OR6C1	SPAG11A
APOC1	CYB5A	IL12RB2	OR6M1	SRP54
ARID1A	DAAM1	IL25	P2RXL1	STAMBP
ARMC6	DAOA	KBTBD4	PAK3	SUCNR1
ATF6B	DCTN4	KCNC2	PAK6	SULT1A1
ATIC	DDX11	KCNH2	PATE3	SYNGR1
ATP1A1	DEFB104A	KIAA1109	PAX1	TLX1
AVP	DEFB113	KIF2B	PDE8B	TMED8
BCAP29	DENND5A	KIF5A	PDLIM5	TMEM225
BTBD8	DKFZP686J0529	KIR3DP1	PFTK2	TPRG1L
BTD	DMGDH	LLGL2	PHF8	TRPM2
C11ORF58	DNAH14	LSR	PKP2	TSLP
C14ORF183	DUOX1	MAP3K3	PLEKHB2	TTC33
C15ORF21	ECSIT	MGC119295	PPM1K	UBXN10
C15ORF44	EEF1A1	MGC33948	PRAMEF22	VWA5A
C1D	EEF1A1	MGLL	PRKAA1	WFDC2
C1ORF185	ERMN	MIDN	PRSS7	XPR1
C1ORF186	EXOD1	MIR1281	PSG6	ZCCHC5
C9ORF47	F5	MPPED1	RAET1L	ZNF302
CCDC64	FAM160A1	MRPL14	RASEF	ZNF562
CCNB1IP1	FAM3B	MRPL43	RGPD4	ZNF598
CCNY	FFAR3	MSH3	RNF39	ZNF611
CD63	FIGLA	MSL3	RPLP0	ZNF66
CD7	FLCN	MYCBP	RPP38	ZNF92
CD86	FLJ37078	NCCRP1	S100A13	ZNRD1
CDC2L5	FLJ45340	NCOA5	SAE1	ZWILCH
CGREF1	FLNC	NEBL	SEC14L1	

Twenty of the 179 cross-talk genes were also found in important GRMs: *DENND5A*, *PAK3*, *IFT80*, *DEFB113*, *STAMBP*, *ERMN*, *DNAH14*, *NRG2*, *EXOD1*, *SHH*, *C1D*, *C9ORF47*, *FIGLA*, *NLRP1*, *XPR1*, *SPAG11A*, *C15ORF44*, *S100A13*, *KIF2B*, and *OR5H14*. These GRMs also included thirteen transcription factors, *AHRR*, *ARID1A*, *ATF6B*, *NCOA5*, *NFIC*, *PAX1*, *SKI*, *TLX1*, *ZNF302*, *ZNF562*, *ZNF598*, *ZNF611*, and *ZNF92*.

### Genes regulated under SHH and EGF Co-stimulation have different biological functions than seen with individual stimulation

Among DRGs under SHH+, we compared the functional enrichment of genes that were already significant under CTRL versus those that became significant only after SHH stimulation (Figure 1). Those already significant genes under CTRL are involved with cell-cycle, cell-division, DNA damage, and DNA repair (Supplementary Figure 2A), while those genes that became significant only after SHH stimulation enriched for terms such as nucleotide-binding, kinases, protein phosphorylation, transcription regulation, and DNA binding (Supplementary Figure 2B). In addition, SHH+ induces transcription regulating activity. Furthermore, we looked at those genes that became silent after the addition of EGF (ie, genes that are DRG under SHH+ but are not DRG under SHH+EGF+ co-stimulation). These genes enriched for transcription factors, mainly the Cys2His2 zinc finger genes (Supplementary Figure 2C).

### Genes identified *in silico* show identical patterns in biological context

**Figure 5:**
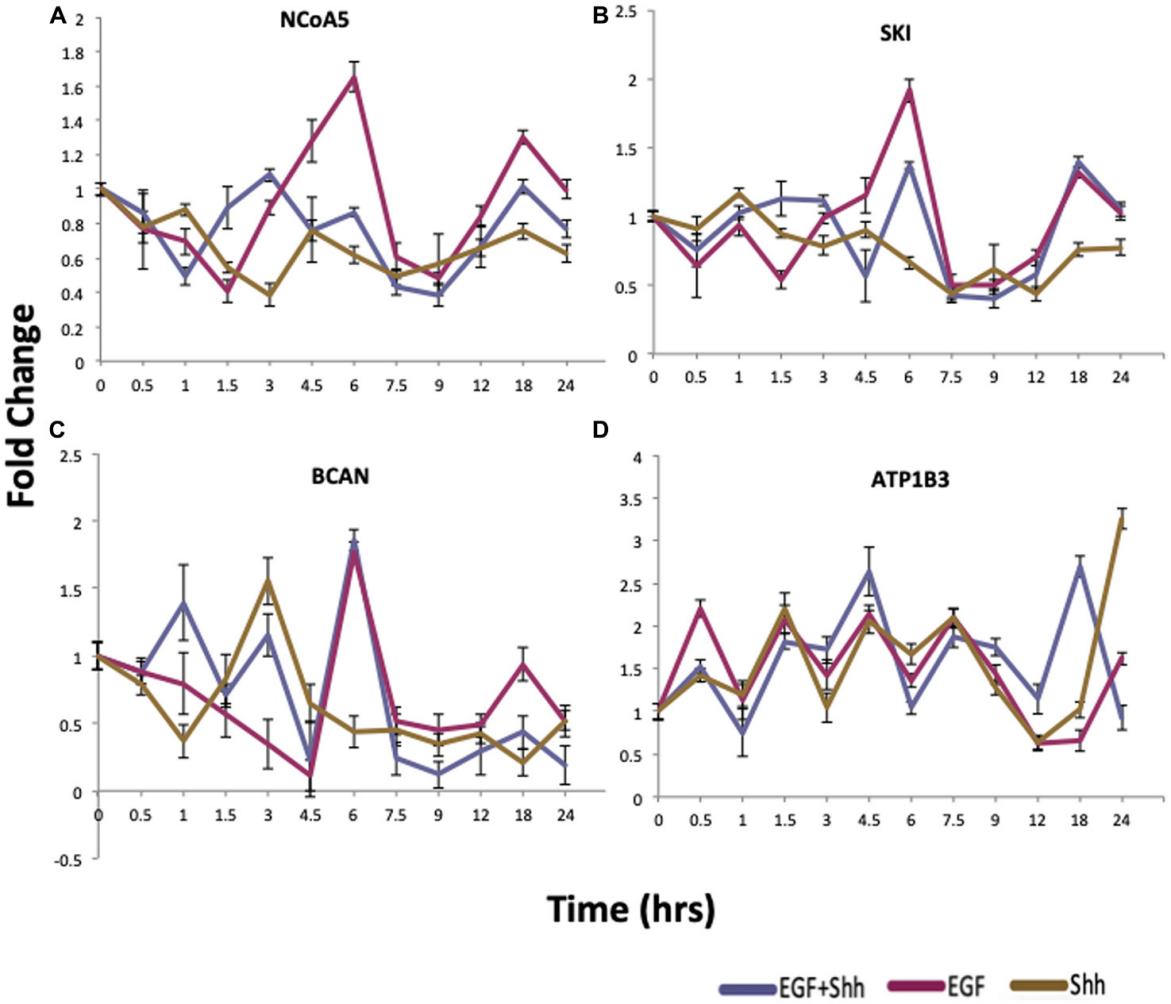
qRT-PCR validation of proposed cross-talk genes. qRT-PCR results of cells treated with EGF (purple), Shh-N conditioned media (gold), or a combinational treatment with Shh-N and EGF (blue). Panels (**A**–**C**) show that EGF dominates the effects of SHH in EGF+SHH treatment after a certain point. Panel (**D**) shows the effect of co-activation, where under EGF+SHH treatment behavior diverges form that under EGF or SHH at and after 12 hours. *n* = 3/group, and data are expressed as mean ± SEM.

To validate the findings from the *in silico* analysis, we performed cell culture experiments according to a previous report [8]. To collect Shh-N conditioned media, HEK293 cells were transfected with Shh-N plasmid and the cell culture supernatant was collected on three consecutive days. The presence of SHH protein in the conditioned media was confirmed by western blotting (Supplementary Figure 3). Then, human medulloblastoma Daoy cells were treated with 4 different conditions: Control, EGF (5 ng/ml), Shh-N conditioned media, and EGF plus Shh-N conditioned media. Cells were collected for quantitative RT-PCR (qRT-PCR) analysis at 14 different time-points over the next 24 hours. We analyzed the expression of a small list of genes presented in Figure 5. Panels (5A–5C) show that, for *NCoA5*, *SKI*, and *BCAN*, activation of EGF dominates the effects of SHH in EGF+SHH treatment after a certain point. Panel 5D confirms the effect of co-activation, where, under EGF+SHH, behavior of *ATP1B3* diverges from that under EGF or SHH after hour 12. We also analyzed *IL8* as a negative control for our *in silico* analysis (Supplementary Figure 4), confirming the synergism results in [8]. As anticipated, the expression pattern of *IL8* did not differ in co-treatment versus any of the single treatments. These results indicate that our *in silico* approach identifies biologically important gene expression patterns and that the results of the *in silico* studies could be validated in a biological setting.

## DISCUSSION

In this study, we implemented an analytic pipeline approach to understand the extent to which SHH and EGF pathways cross-talk. We re-analyzed publicly available data using a well-developed computational pipeline, and it led to several compelling results. In order to compare our results with those in a previous report [8], we must keep in mind that we were not aiming to find quantitative synergistic effects, rather, our focus was on trends in how expression patterns change over time. For this reason, the main findings of the previous report on the synergistic effects of selective genes *GLI*, *PTCH*, *HHIP*, *MMP7*, *VEGFA*, and *IL8* did not show up in our cross-talk list as they were only distinguished by quantitative comparison of expression level. In contrast, the genes identified and validated in this study (Figure 5) might not appear when using conventional methods, because their expression was not significantly changed if one were to only compare the initial and final conditions. This illustrates the value of our method because these genes might be important of cell function and cancer progression.

The power of statistical hypothesis tests that target differences in quantity is highly dependent on sample size. For a large enough sample size, even an ignorable difference in values might be presented as a significant change. Such a statistical limitation might partly affect our methodology as well, especially where we used a statistical F-test to identify DRGs. Seven to 8 time points are required at minimum for the methodology to be valid. The dataset analyzed here has 14 time points, more than enough to identify trends. However, due to this requirement, we were limited to only studying the dataset used here because there was no other cell line or patient datasets that met this requirement. This is one limitation of the current study.

We explored the expression profile of all genes under four different conditions: CTRL, SHH+, EGF+, and SHH+EGF+. Under the CTRL condition, as we expected, genes related to regular cell cycle activities were dynamically active. Due to the artificial condition of cell culture in 24 hours, the genes in CTRL showed various expression patterns. Some genes decreased expression (GRM_C2), and some genes temporally increased expression before decreasing (GRM_C3), probably due to the depletion of nutrients in the culture media. The expression of other genes involved in cell signaling or stress responses were increased in various patterns (GRM_C1, C4, and C5). Our enrichment analysis also detected that SHH+ stimulus induced a response through transcriptional changes. In addition, we identified genes that become dynamically silent upon co-stimulation, and these genes were generally transcriptional factors. This is a novel finding and the genes identified in this study have the potential to be explored further as therapeutic targets. Furthermore, we showed the extent of the influence of SHH+ and EGF+ on the dynamic activity of the gene population. EGF+ has considerably greater influence than SHH+ in modifying the activity of genes, and during co-stimulation, EGF+ plays a dominant role, overshadowing most of the SHH effects as seen by the high correlation between the EGF+ and SHH+EGF+ expression profiles for most genes.

During co-stimulation, several genes altered their expression profile in a manner that was not similar to that observed under SHH+ or EGF+. These cross-talk genes can be used as markers to study or test for the co-stimulation effects of EGF in an SHH+ environment. These genes could also be new targets for cancer treatment. Five of these genes (*AHHR*, *ARID1A*, *NCOA5*, *NFIC*, and *SKI*) [19–23] have been determined to be tumor suppressors in major studies, and 13 of them are transcription factors (*AHRR*, *ARID1A*, *ATF6B*, *NCOA5*, *NFIC*, *PAX1*, *SKI*, *TLX1*, *ZNF302*, *ZNF562*, *ZNF598*, *ZNF611*, and *ZNF92*). The genes that were further validated by biological experiments were selected solely on the basis of the correlation measures in the initial screening and not by their biologic importance nor by their influence in cancer progression. However, a quick literature search revealed that they have been a focus of studies by many researchers for their significant role in progression of various types of cancer. Specifically, Gao et al. [21] showed that reduced *NCOA5* expression is associated with a fraction of human hepatocellular carcinoma cancers (HCC) and argued that susceptibility to both glucose intolerance and HCC increases with *NCOA5* deficiency. Li et al. [24] reported higher expression of ATP1B3 protein in gastric cancer tissues than in normal matched tissues, and its knockdown significantly inhibits cell proliferation, colony-formation ability, migration, and invasion, and also increases apoptosis in human gastric carcinoma cell lines. BCAN (Brevican) has been used as a target for immunotherapy in glioma, as its knockdown reduced late-stage glioma tumor aggressiveness [25, 26]. CMAS, which is in the sialic acid pathway, has a key role in breast cancer and is significantly associated with decreased breast cancer patient survival. Also, low CMAS gene expression is correlated with reduced recurrence-free survival in a human colorectal cancer cohort [27, 28]. XCL2 expression increases with the degree of malignancy of lung cancer, indicating that it could be an important target in gene therapy for lung cancer [29]. SKI has been described as a potent negative regulator of TGF beta signaling [30]. Although upregulation of *SKI* has been reported in many cancers such as colorectal cancer, it is labeled as a tumor suppressor by many studies. *SKI* was also identified as a target gene of the hematopoietic transcription factor c-*MYB*, which is involved in the proliferation and differentiation of progenitor cells of myeloid and lymphoid lineages. NRG2 and PAK3 belong to ErbB signaling pathway. *NRG2* clustered with *DNAH14*, also a cross-talk gene, in a GRM with high in-degree (Regulated by 18% of all GRMs), while *PAK3* clustered with *DENND5A*, another cross-talk gene, in a GRM that was important with respect to out-degree (Regulating 36% of all GRMs). NRGs belong to the large family of EGF ligands, and are implicated in brain development activities by interacting with ErbB [31] and regulating HER2, HER3, and HER4 (ERBB2, ERBB3 and ERBB4, respectively, in mice), all of which have been linked to different types of cancers in the literature [32].

Our statistical and computational methods have been tested and confirmed via multiple simulation studies. In addition, in this study, we validated our *in silico* results with cell culture experiments in a biological context. These results indicate that our approach has the power to identify novel therapeutic targets from publicly available datasets in a more credible manner than conventional methods.

## MATERIALS AND METHODS

### Expression profile data

In this study, we re-analyzed the experimental data in which, human medulloblastoma cell lines were exposed to four experimental conditions (CTRL, SHH+, EGF+, and SHH+EGF+) and sampled over 24 hours after the exposure to the agents. Gene expression data was generated with an Illumina microarray platform. There were three biological replicates available and we chose to take the median of the replicates for the analysis, since it is robust to unexpected variations. The fold-change ratios were calculated with respect to time 0 under the control. Further details of the experimental materials and methods are described in the literature [8]. The data set is available on the GEO website with the series name GSE46045.

### Data analysis

For our analysis, we used a pipeline developed in our previous report [3]. There are three main steps: First, dynamic response genes (DRG), ie, genes that exhibit significant changes over time, were identified by statistical hypothesis testing. Then, the DRGs were clustered into gene response modules (GRM), clusters of one or many genes that follow a similar expression pattern over time. Finally, a gene regulatory network (GRN) between the GRMs was built by using ordinary differential equations. In addition, we used Spearman correlation to study the differences in the expression profile among genes or GRMs under the four experimental conditions.

### Identification of the Dynamic Response Genes (DRG)

Suppose the centered expression profile of the gene, *ith* belonging to subject $j,X_{i,j}\left(t\right)$, is a smooth function over time, and the time course gene expression measurements are discrete observations from this smooth function, which have been distorted by noise, i. e.,$Y_{i,j}\left(t_{k}\right)-\mu_{i,j}=X_{i,j}\left(t_{k}\right)+E_{i,j}\left(t_{k}\right)$, for i=1,…,n,j=1,…,N and $\text{k}=1,\ldots ,K_{i,j}$, where n is the number of genes, N is the number of subjects, and $K_{i,j}$ is the number of time points observed for the ith gene, belonging to subject j. The noise denoted by $E_{i,j}\left(t_{k}\right)$ is assumed to be independently and identically distributed (i.i.d.) with mean 0 and variance $\sigma^{2}$. The pipeline obtains the functional entity $X_{i,j}\left(t\right)$ by spline smoothing [33]. It chooses a subset of the genes that exhibit time course patters that have relatively smooth trajectories that do not fluctuate widely. Then these genes are ranked by their interquartile range and select the top genes for the estimation subset. It is reasonable to include only these responsive genes in the analysis, since many genes have a flat trajectory and so not carrying any information; hence the pipeline uses the following statistical hypothesis testing to identify DRGs.

$$\begin{array}{ccc} H_{0}:X_{i,j}\left(t\right)\equiv 0 & vs & H_{a}:X_{i,j}\left(t\right) \end{array}≢0$$

### Clustering DRGs into GRMs

As many of the DRGs exhibit similar expression patterns over time we clustered them into temporal gene response modules by using the Iterative Hierarchal Clustering (IHC) method. IHC identifies inhomogeneous clusters, captures both the large and very small clusters, and provides an automated selection of the optimal number of clusters. This step is biologically interpretable as genes tend to act in collaboration with each other, and for computational purposes, it reduces the size and dimensionality of the problem.

### Discovery of the high-dimensional Gene Regulation Network (GRN) by using differential equation models

A gene regulatory network attempts to map how different genes control the expression of other genes. The gene regulations can be modeled by rate equations,

$$\begin{array}{ccc} DM_{q,j}=\alpha_{0,q,j}+\sum_{p=1}^{Q}\alpha_{p,q,j}M_{p,j} & for & q=1,2,\ldots ,Q, \end{array}$$

where $\alpha_{0,q,j}$ is the intercept for the $q^{th}$ gene response module, belonging to the subject j, and the coefficients ${\left\{M_{p,q,j}\right\}}_{p=1}^{Q}$ quantify the regulation effects of the $p^{th}$ gene response module on the instantaneous rate of change in $q^{th}$ gene response module. This model can appropriately capture both up and down regulation as well as up and down self-regulation. Typically, only a few gene response modules will affect the instantaneous rate of change in $q^{\text{th}}$ gene response module, thus only a few of the ${\left\{M_{p,q,j}\right\}}_{p=1}^{Q}$ will be non-zero. We first perform a model selection that determines which ${\left\{M_{p,q,j}\right\}}_{p=1}^{Q}$ are non-zero and then we estimate their coefficients to determine the regulation effects. Here we use the ordinary differential equation (ODE) modelling approach in order to reconstruct the high-dimensional GRN [34–36].

### Comparisons

The pipeline uses the Spearman correlation between the expression values of two genes to measure the level of their similarity, a measure which is also exploited for the clustering purposes in Section 4.3. A large positive correlation indicates small or no change, while a large negative value and small correlation close to zero (in absolute value) implies significant change in the temporal behavior of the gene under two different conditions. Supplementary Figure 5 demonstrates three selected genes: *HLA-DMB*, *KHDC1*, and *ZC3HAV1* under two different conditions (CTRL and EGF) with correlations –0.92, 0.94, 0.002.

### Cross-talk algorithm, notations, and definitions

A gene is said to have differential activity under condition 1 compared to condition 2, if the Spearman correlation between the mean curve under the two conditions is less than 0.7.

“Important GRMs” are defined as those GRMs with at least one of their In-degree, Out-degree, and Betweenness coefficients in the 95th percentile. For each GRM (as a vertex in the regulatory network), In-degree is the number of the GRMs regulating that GRM. Out-degree is the number of GRMs regulated by that GRM, and Betweenness quantifies the number of times it acts as a bridge along the shortest path between two other nodes. For node *ν* it is calculated as

$$Bet\left(v\right)=\sum_{s\neq v}\frac{\sigma_{st}\left(v\right)}{\sigma_{st}}$$

Where, $\sigma_{st}$ is total number of shortest paths from node*s* to node t and $\sigma_{st}\left(v\right)$ is the number of those paths that pass through v.

An expression profile over time is said to have “significant variation” if its range of variation is not smaller than 1.96. The critical value 1.96 is motivated by the significance critical value of a normal distribution at the 0.05 level.

Cross-Talk expression pattern: where, the expression curve shows;

- Differential activity under SHH+ vs CTRL
- Significant variation under SHH
- Differential activity under EGF+ vs CTRL
- Significant variation under EGF+
- Differential activity under EGF+SHH+ vs SHH+
- Differential activity under EGF+SHH+ vs EGF+

Co-activation expression pattern: where, the expression curve shows;

- No differential activity under SHH+ vs CTRL
- No differential activity under EGF+ vs CTRL
- Differential activity under EGF+SHH+ vs SHH+

### Cell culture

Daoy cells (ATCC: HTB-186) and HEK293T cells were cultured in Dulbecco’s modified Eagle’s medium (DMEM) containing 10% fetal bovine serum (FBS) and 1% antibiotics. Hyper-confluent Daoy cells were pre-starved for 24 hours in DMEM plus 0.5% FBS, and then were treated with EGF (5 ng/ml), Shh-N conditioned media (50% v/v), a combination of both, or a control media. For the SHH enriched condition medium preparation, HEK293T cells were transfected with a Shh-encoding plasmid (37680, Addgene) by using lipofectamine 2000 (ThermoFisher) [21]. The cell culture supernatant was collected every 24 hours for 3 days, centrifuged, and the supernatant was prepared as a condition media and stored in –80°C. To monitor the SHH synthesis and presence in the harvested conditioned media, western blots were carried out with 30 ul of conditioned media.

### qRT-PCR analysis

Total RNAs were extracted from Daoy cells with an RNA STAT-60 (Tel-Test) and RNeasy mini kit (QIAGEN), according to the manufacturer protocols. The quantity and purity of the RNA were determined by using a NanoDrop spectrophotometer (ThermoFisher). Total RNA was treated with DNase I, followed by cDNA synthesis with reverse transcriptase and random hexamers. Quantitative RT-PCR reactions were performed with 25 ng cDNA, 150 nM of each primer, and SYBR Green PCR Master Mix (Invitrogen) in triplicate by using a Quant Studio 6 Flex instrument (Applied Biosystems). Relative mRNA levels were calculated by using the comparative CT method normalized to cyclophilin. The primers were designed using Primer Express Software (Applied Biosystems) as shown in Table 7.

**Table 7:** Primer sequences used for qRT-PCR analysis

Gene	Forward Primer	Reverse Primer
***ATP1B3***	TTC CAA AAC CAG TGA CCG CAT TG	AGT GCT CCA TCA GGA CAG ACT G
***BCAN***	TAA GCA CAG CCG CTT CAA CGT C	CTC TGT CAC TGT GAC GAT AGC C
***NCoA5***	AGA TTC ACC GCT CCT GCA CAG T	CTG TCT GGC AAT CTC CTC ACG T
***SKI***	CCT TCC GAA AAG GAC AAG CCG T	GCT CTT TCT CAC TCG CTG ACA C
***IL8***	GAG AGT GAT TGA GAG TGG ACC AC	CAC AAC CCT CTG CAC CCA GTT T
***Cyclophilin***	GGA GAT GGC ACA GGA GGA A	GCC CGT AGT GCT TCA GTT T

## SUPPLEMENTARY MATERIALS



## Abbreviations

SHH: Sonic hedge hog
EGF: Epidermal growth factor
ANOVA: Analysis of covariance
ODE: Ordinary differential equation
DGEs: Dynamic response genes
GRMs: Gene response modules
FBS: Fetal bovine serum
ATP1B3: ATPase Na+/K+ transporting subunit beta 3
NCoA5: Nuclear receptor coactivator 5
SKI: SKI proto-oncogene
BCAN: Brevican
IL-8: Interleukin-8
