# Supplementary material for: Gene expression dynamic analysis reveals co-activation of Sonic Hedgehog and epidermal growth factor followed by dynamic silencing
Source: Oncotarget. 2020 Apr 14;11(15):1358–72. doi: 10.18632/oncotarget.27547 (PMC7170495; doi:10.18632/oncotarget.27547)
Supplement: Supplementary file 1 [file oncotarget-11-1358-s001.pdf]

Gene expression dynamic analysis reveals co-activation of Sonic Hedgehog and epidermal growth factor followed by dynamic silencing

SUPPLEMENTARY MATERIALS

Western blot

The samples were loaded on an SDS-PAGE gel and run at 110V for 1hr. After transferring the proteins to a PVDF membrane, the membrane was blocked with 5% milk solution, followed by incubation with primary antibody (anti-Shh, C9C5, Cell Signaling, 1:1000) overnight at 4°C. The membrane was washed 3 times with TBS-Tween 20 and incubated with an anti-rabbit IgG HRP-linked antibody (Cell Signaling, 7074, 1:2000) for 1 hour at RT. Then, the membrane was washed 3 times with TBS-Tween 20, developed with chemiluminescence substrate (ThermoFisher), and images were captured with an Odyssey Fc imaging system (LI-COR).

GRM_C1

Annotation Cluster 1	Enrichment Score: 1.93	G	
UP_KEYWORDS	Ubl conjugation pathway	RT	
GOTERM_MF_DIRECT	ubiquitin-protein transferase activity	RT	
GOTERM_BP_DIRECT	protein ubiquitination	RT	
Annotation Cluster 2	Enrichment Score: 1.83	G	
UP_KEYWORDS	Zinc-finger	RT	
UP_KEYWORDS	Zinc	RT	
UP_KEYWORDS	Metal-binding	RT	
GOTERM_MF_DIRECT	metal ion binding	RT	
Annotation Cluster 3	Enrichment Score: 1.68	G	
UP_KEYWORDS	Wnt signaling pathway	RT	
GOTERM_BP_DIRECT	negative regulation of canonical Wnt signaling pathway	RT	
GOTERM_BP_DIRECT	Wnt signaling pathway	RT	

GRM_C2

Annotation Cluster 1	Enrichment Score: 6.46	G	
UP_KEYWORDS	Cell cycle	RT	
UP_KEYWORDS	Mitosis	RT	
UP_KEYWORDS	Cell division	RT	
GOTERM_BP_DIRECT	cell division	RT	
GOTERM_BP_DIRECT	mitotic nuclear division	RT	
Annotation Cluster 2	Enrichment Score: 6.03	G	
GOTERM_BP_DIRECT	DNA replication	RT	
UP_KEYWORDS	DNA replication	RT	
KEGG_PATHWAY	DNA replication	RT	
GOTERM_BP_DIRECT	G1/S transition of mitotic cell cycle	RT	
GOTERM_BP_DIRECT	DNA replication initiation	RT	
GOTERM_BP_DIRECT	telomere maintenance via recombination	RT	
GOTERM_CC_DIRECT	alpha DNA polymerase:primase complex	RT	
GOTERM_BP_DIRECT	DNA strand elongation involved in DNA replication	RT	
KEGG_PATHWAY	Pyrimidine metabolism	RT	
GOTERM_MF_DIRECT	DNA primase activity	RT	
GOTERM_BP_DIRECT	DNA replication, synthesis of RNA primer	RT	
KEGG_PATHWAY	Purine metabolism	RT	
GOTERM_MF_DIRECT	DNA-directed DNA polymerase activity	RT	
UP_KEYWORDS	Nucleotidyltransferase	RT	
Annotation Cluster 3	Enrichment Score: 5.06	G	
UP_KEYWORDS	DNA damage	RT	
UP_KEYWORDS	DNA repair	RT	
GOTERM_BP_DIRECT	DNA repair	RT	
GOTERM_MF_DIRECT	damaged DNA binding	RT	

GRM_C3

Annotation Cluster 1	Enrichment Score: 7.02	G
UP_KEYWORDS	Cell cycle	RT
UP_KEYWORDS	Cell division	RT
UP_KEYWORDS	Mitosis	RT
GOTERM_BP_DIRECT	cell division	RT
GOTERM_BP_DIRECT	mitotic nuclear division	RT
**Annotation Cluster 2**	**Enrichment Score: 2.95**	**G**
UP_KEYWORDS	Nucleotide-binding	RT
UP_KEYWORDS	ATP-binding	RT
GOTERM_MF_DIRECT	ATP binding	RT
UP_SEQ_FEATURE	nucleotide phosphate-binding region:ATP	RT
INTERPRO	P-loop containing nucleoside triphosphate hydrolase	RT
**Annotation Cluster 3**	**Enrichment Score: 2.38**	**G**
GOTERM_BP_DIRECT	DNA replication	RT
UP_KEYWORDS	DNA replication	RT
KEGG_PATHWAY	DNA replication	RT
KEGG_PATHWAY	Mismatch repair	RT
GOTERM_BP_DIRECT	telomere maintenance via recombination	RT
KEGG_PATHWAY	Nucleotide excision repair	RT
GOTERM_BP_DIRECT	nucleotide-excision repair, DNA gap filling	RT
GOTERM_BP_DIRECT	transcription-coupled nucleotide-excision repair	RT
GOTERM_BP_DIRECT	nucleotide-excision repair, DNA incision, 5'-to lesion	RT
GOTERM_BP_DIRECT	nucleotide-excision repair, DNA incision	RT

GRM_C4

Annotation Cluster 1	Enrichment Score: 1.98	G
UP_KEYWORDS	Chaperone	RT
GOTERM_BP_DIRECT	protein folding	RT
GOTERM_MF_DIRECT	chaperone binding	RT
GOTERM_MF_DIRECT	unfolded protein binding	RT
**Annotation Cluster 2**	**Enrichment Score: 1.85**	**G**
UP_SEQ_FEATURE	domain:Fibronectin type-III 2	RT
UP_SEQ_FEATURE	domain:Fibronectin type-III 1	RT
UP_SEQ_FEATURE	domain:Fibronectin type-III 5	RT
UP_SEQ_FEATURE	domain:Fibronectin type-III 4	RT
UP_SEQ_FEATURE	domain:Fibronectin type-III 3	RT
**Annotation Cluster 3**	**Enrichment Score: 1.69**	**G**
SMART	KH	RT
INTERPRO	K Homology domain	RT
INTERPRO	K Homology domain, type 1	RT
GOTERM_BP_DIRECT	negative regulation of translation	RT
UP_SEQ_FEATURE	domain:KH 1	RT
UP_SEQ_FEATURE	domain:KH 2	RT
GOTERM_MF_DIRECT	mRNA binding	RT
GOTERM_CC_DIRECT	polysome	RT

GRM_C5

Annotation Cluster 1	Enrichment Score: 3.66	G	
UP_KEYWORDS	Stress response	RT	
INTERPRO	Heat shock protein 70, conserved site	RT	
INTERPRO	Heat shock protein 70 family	RT	
Annotation Cluster 2	Enrichment Score: 3.35	G	
UP_KEYWORDS	Stress response	RT	
GOTERM_BP_DIRECT	response to unfolded protein	RT	
UP_KEYWORDS	Chaperone	RT	
GOTERM_MF_DIRECT	unfolded protein binding	RT	
Annotation Cluster 3	Enrichment Score: 1.07	G	
UP_KEYWORDS	ATP-binding	RT	
GOTERM_MF_DIRECT	ATP binding	RT	
UP_KEYWORDS	Nucleotide-binding	RT	

Supplementary Figure 1: Enrichment cluster analysis of the LS-GRM in the CTRL group. Enrichment cluster analysis of 5 major LS-GRM genes was performed by using the DAVID functional annotation clustering tool. For each GRM, the first three major clusters are presented here.

A Genes of DRG under both CTRL and SHH conditions.

Annotation Cluster 1	Enrichment Score: 13.97	G
UP_KEYWORDS	Cell cycle	RT
UP_KEYWORDS	Cell division	RT
UP_KEYWORDS	Mitosis	RT
GOTERM_BP_DIRECT	cell division	RT
GOTERM_BP_DIRECT	mitotic nuclear division	RT
Annotation Cluster 2	Enrichment Score: 5.58	G
UP_KEYWORDS	DNA damage	RT
UP_KEYWORDS	DNA repair	RT
GOTERM_BP_DIRECT	DNA repair	RT
Annotation Cluster 3	Enrichment Score: 3.71	G
GOTERM_BP_DIRECT	sister chromatid cohesion	RT
UP_KEYWORDS	Centromere	RT
UP_KEYWORDS	Kinetochore	RT
GOTERM_CC_DIRECT	condensed chromosome kinetochore	RT

B Genes silent under CTRL and DRG under SHH.

Annotation Cluster 1	Enrichment Score: 2.9	G
UP_KEYWORDS	Nucleotide-binding	RT
UP_KEYWORDS	ATP-binding	RT
UP_SEQ_FEATURE	domain:Protein kinase	RT
UP_SEQ_FEATURE	nucleotide phosphate-binding region:ATP	RT
UP_KEYWORDS	Kinase	RT
INTERPRO	Protein kinase, catalytic domain	RT
UP_SEQ_FEATURE	binding site:ATP	RT
UP_SEQ_FEATURE	active site:Proton acceptor	RT
INTERPRO	Protein kinase-like domain	RT
UP_KEYWORDS	Transferase	RT
GOTERM_MF_DIRECT	protein kinase activity	RT
GOTERM_MF_DIRECT	ATP binding	RT
UP_KEYWORDS	Serine/threonine-protein kinase	RT
SMART	S_TKc	RT
INTERPRO	Serine/threonine-protein kinase, active site	RT
GOTERM_MF_DIRECT	protein serine/threonine kinase activity	RT
INTERPRO	Protein kinase, ATP binding site	RT
GOTERM_BP_DIRECT	protein phosphorylation	RT
Annotation Cluster 2	Enrichment Score: 2.49	G
UP_SEQ_FEATURE	repeat:WD 6	RT
UP_KEYWORDS	WD repeat	RT
INTERPRO	WD40 repeat	RT
UP_SEQ_FEATURE	repeat:WD 1	RT
UP_SEQ_FEATURE	repeat:WD 2	RT
UP_SEQ_FEATURE	repeat:WD 3	RT
UP_SEQ_FEATURE	repeat:WD 5	RT
SMART	WD40	RT

C Genes of DRG under SHH but silent under EGF+SHH+.

Annotation Cluster 1	Enrichment Score: 2.99	G	
UP_KEYWORDS	DNA-binding	RT	
UP_KEYWORDS	Transcription regulation	RT	
UP_KEYWORDS	Transcription	RT	
GOTERM_BP_DIRECT	transcription, DNA-templated	RT	
GOTERM_MF_DIRECT	DNA binding	RT	
INTERPRO	Zinc finger, C2H2-like	RT	
INTERPRO	Zinc finger, C2H2	RT	
GOTERM_BP_DIRECT	regulation of transcription, DNA-templated	RT	
UP_KEYWORDS	Nucleus	RT	
SMART	ZnF_C2H2	RT	
GOTERM_CC_DIRECT	nucleus	RT	
Annotation Cluster 2	Enrichment Score: 2.3	G	
UP_KEYWORDS	Cytokine	RT	
KEGG_PATHWAY	Cytokine-cytokine receptor interaction	RT	
GOTERM_MF_DIRECT	cytokine activity	RT	
Annotation Cluster 3	Enrichment Score: 2.03	G	
UP_SEQ_FEATURE	glycosylation site:N-linked (GlcNAc...)	RT	
UP_KEYWORDS	Glycoprotein	RT	
UP_SEQ_FEATURE	signal peptide	RT	
UP_SEQ_FEATURE	disulfide bond	RT	
UP_KEYWORDS	Disulfide bond	RT	
UP_KEYWORDS	Signal	RT	

Supplementary Figure 2: Enrichment cluster analysis for subsets of genes with different behavior under different conditions. Enrichment cluster analysis of 3 lists of genes was performed by using the DAVID functional annotation clustering tool. (**A**). Genes that are active (DRGs) under CTRL and SHH; (**B**) Genes that are silent under CTRL but are activated under SHH+; (**C**). Genes that are DRGs under SHH+ but silent under EGF+SHH+. For each GRM, the first two to three major clusters are presented here.

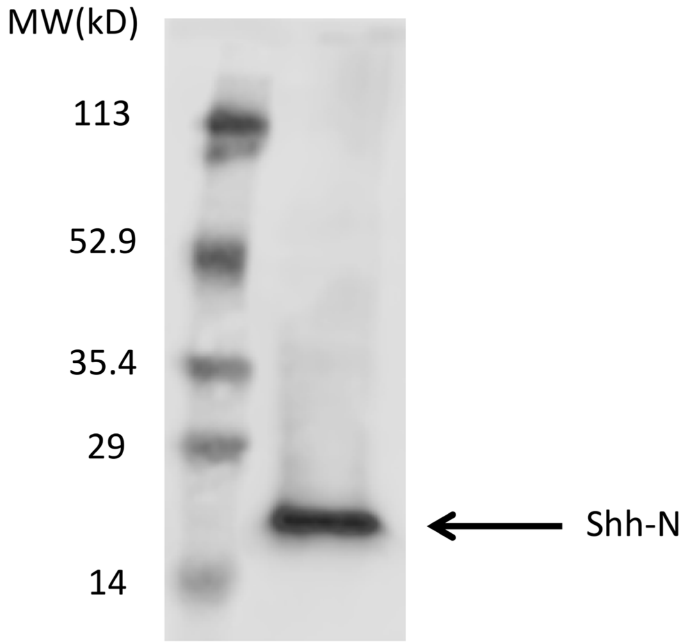

Supplementary Figure 3: Western blot of SHH proteins. The western blot of the conditioned media collected after transfecting HEK293 cell with Shh-N plasmid.

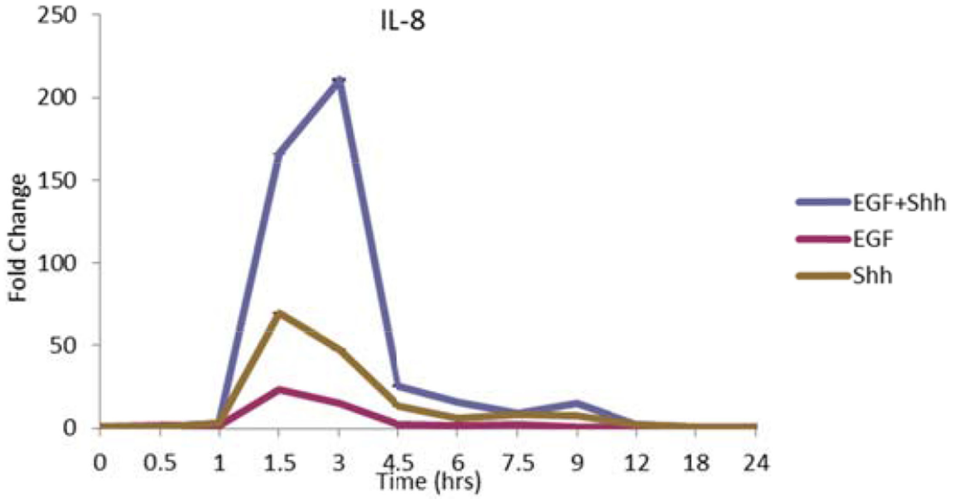

Supplementary Figure 4: qRT-PCR validation of the *IL8* gene expression. This figure confirms the synergism between EGF and SHH similar to the Götschel, F., et al. paper.

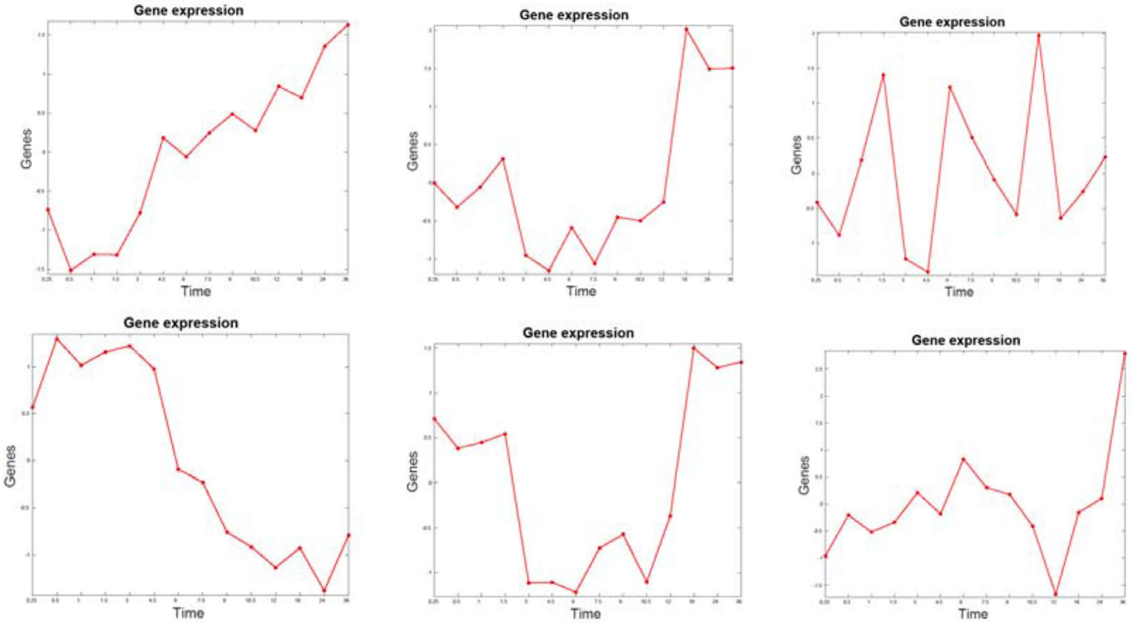

Supplementary Figure 5: Spearman correlation and behavior similarity. Each column presents the time-resolved behavior of one of the three genes (respectively, from left to right, *HLA-DMB*, *KHDC1*, and *ZC3HAV1*) under two conditions: control (top row) and EGF (bottom row). The corresponding correlations between the two curves in each column are $\rho = -0.92$, 0.94, and 0.002, respectively. High negative (positive) values represents opposite (similar) behaviors under the two conditions.
